# Treatment of Knee Osteoarthritis and Chondral Injury with Umbilical Cord/Wharton’s Jelly-Derived Mesenchymal Stem Cells: A Systematic Review of Safety and Efficacy

**DOI:** 10.3390/jfb16030084

**Published:** 2025-03-01

**Authors:** Mohd Ishak-Samrin, Isa Naina-Mohamed, Mohamed S. Zulfarina, S. Fadilah Abdul Wahid, Ahmad Farihan Mohd Don, Norlelawati Mohamad, Muhamad Karbela Reza Ramlan, Akmal Hisham Md Yusoff Badrul

**Affiliations:** 1 Department of Orthopaedic and Traumatology, Faculty of Medicine, Hospital Canselor Tuanku Muhriz, Universiti Kebangsaan Malaysia, Kuala Lumpur 56000, Malaysia; ishaque89@gmail.com (M.I.-S.);; 2 Pharmacoepidemiology and Drug Safety Unit, Department of Pharmacology, Faculty of Medicine, Hospital Canselor Tuanku Muhriz, Universiti Kebangsaan Malaysia, Kuala Lumpur 56000, Malaysia; 3 Cell Therapy Center, Hospital Canselor Tuanku Muhriz, Universiti Kebangsaan Malaysia, Kuala Lumpur 56000, Malaysia

**Keywords:** knee osteoarthritis, chondral injury, umbilical cord, Wharton’s jelly, mesenchymal stem cells

## Abstract

Knee osteoarthritis (KOA) is a major cause of pain and disability worldwide, with no current treatment modality able to prevent the progressive destruction of articular cartilage. Mesenchymal stem cells (MSCs) have attracted interest in treating KOA and cartilage injury due to their self-renewal and multidirectional differentiation potential, as well as local bioactive factors with immunomodulatory and regenerative potential. This study aimed to evaluate the currently available studies using the intra-articular injection of Wharton’s jelly MSCs for KOA and cartilage injury. We analyzed all clinical trials published from inception to 31 December 2023. Six studies met the inclusion criteria, for a total of 97 patients and 134 knees. The follow-up period ranged from 3 to 48 months. There were no serious adverse effects noted. There was significant improvement in functional outcomes in the form of VAS, WOMAC, KOOS, and IKDC parameters, though radiological outcomes demonstrated mixed results. In conclusion, patients with KOA treated with intra-articular injections of Wharton’s jelly MSCs showed improvements in functional outcomes with no severe adverse effects. Multiple injections of Wharton’s jelly MSCs showed better outcomes compared to single-injection techniques. Wharton’s jelly MSCs may have potential as a cornerstone in the future treatment of KOA.

## 1. Introduction

Knee osteoarthritis (OA) is one of the most common joint disorders in the world, with an estimated 251 million people suffering from knee OA worldwide, according to the Global Burden of Disease 2010 Study. OA is also more prevalent in the aging population. The World Health Organization (WHO) estimated that 524 million people suffering from OA in 2010 were aged 65 or older, and this number is expected to triple, representing 16% of the world’s population by 2050. Knee OA is characterized by the progressive loss of articular cartilage, osteophytes, subchondral cysts, joint space narrowing, and intermittent inflammation of the joint tissues [1]. The exact pathogenesis of OA is still poorly understood, but it is thought to be a complex interplay among mechanical, biochemical, cellular, genetic, and immunologic phenomena [2]. The results of the progressive loss of joint cartilage may suggest there is an imbalance between the rate of cartilage loss and restoration, which may be due to the failure of the body to replenish the cartilage lost through normal use and aging, an inadequate response to injury or inflammation, or both [3]. On a related issue, articular cartilage injury of the knee may result from acute traumatic injury or subacute injury associated with other knee pathologies. Articular cartilage demonstrates limited regenerative potential in response to injury and, therefore, has been implicated as a potential risk factor in the development of early-onset OA [4].

According to the American Academy of Orthopaedic Surgeons (AAOS), the current treatment modalities for knee OA range from non-surgical methods, which include lifestyle modification, physiotherapy, multimodal analgesia, and intra-articular knee injection, to operative options, which include arthroscopic procedures, corrective osteotomies, and joint replacement surgeries [5]. The treatments for knee chondral injury range from symptomatic treatment with the usage of multimodal analgesia, intra-articular knee injections to surgical options, which include surgical debridement, cartilage reparative procedures including marrow stimulation techniques, cartilage replacement such as osteochondral transplantation, or regeneration for example autologous chondrocyte implantation.

While some of the treatment options may benefit patients through mainly symptomatic relief, none of them can prevent the affected articular cartilage from progressive destruction [6]. Interest in the use of biologic agents for regenerative medicine applications including in knee OA and chondral injury has increased in recent decades. The usage of mesenchymal stem cells (MSCs) in cartilage repair was pioneered by Wakitani et al. in 1998 [7]. The exact mechanism through which stem cell therapy may benefit knee OA is still not fully understood. Although native articular cartilage has a limited ability to regenerate, it is believed that injecting an adequate number of stem cells into the affected joint could alleviate symptoms or improve joint structure and function. This improvement may occur through cartilage restoration, stimulation of the body’s natural repair processes via growth factor secretion, support of other repair cells, or modulating the immune response [8].

There are multiple sources of MSCs, including bone marrow, adipose tissue, umbilical cord including Wharton’s jelly (WJ), amniotic fluid, blood, liver, and dental tissues. WJ is a mucoid connective tissue enclosing the three blood vessels of the umbilical cord, which is inherently rich with mesenchymal stem cells. WJ offers multiple distinct advantages, which include [9] (1) properties from both prenatal and postnatal MSCs; (2) no ethical issues regarding acquisition; (3) strong potential for proliferation and differentiation; (4) no risk of tumorigenicity; (5) stability of the karyotype; (6) elevated immunomodulatory activity; and (7) for patients in whom autologous MSCs have weaker stem cell capability.

Currently, the systematic reviews that concentrate on human umbilical cord (hUC) MSCs from WJ sources are limited. This systematic review was conducted to evaluate the current data regarding the safety and efficacy of hUC-MSCs from WJ sources for the treatment of knee OA and chondral injury.

## 2. Materials and Methods

### 2.1. Search Method

For a comprehensive search of health science journals, we used CINAHL (via EBSCOhost) and Medline (via PUBMED). The database was searched until 31 December 2023. The search strategy included all research trials using terms and keywords with Boolean operator strategies. The terms used were Wharton jelly, umbilical cord, mesenchymal stem cell, osteoarthritis, chondral, cartilage, knee, pain, function, QOL, quality of life, KOOS, IKDC, WOMAC, and Lysholm score, as listed below:

Keyword 1 “Wharton jelly” OR “umbilical cord” OR “mesenchymal stem cell” OR “osteoarthritis” OR “chondral” OR “cartilage” AND “knee”

AND

Keyword 2 “Pain” OR “function” OR “QOL” OR “quality of life” OR “KOOS” OR “IKDC” OR “WOMAC” OR “Lysholm”

Furthermore, the references of all retrieved articles were reviewed for relevant citations.

### 2.2. Inclusion Criteria

All human clinical trials that investigated the effect of hUC-MSCs from WJ sources in knee OA and cartilage injury were included from inception until 31 December 2023. Due to limited resources, only manuscripts written in English were included in this review.

### 2.3. Exclusion Criteria

Case reports, case series, animal studies, letters to the editor, posters, and review articles were excluded.

### 2.4. Screening of Article for Eligibility

We conducted this systematic review according to the Preferred Reporting Items for Systematic Review and Meta-Analyses (PRISMA) guidelines. This systematic review was registered with the Open Science Framework Database (https://osf.io) on 25 August 2024 and possesses a registration DOI of https://doi.org/10.17605/OSF.IO/P5NHY.

**Figure 1:**
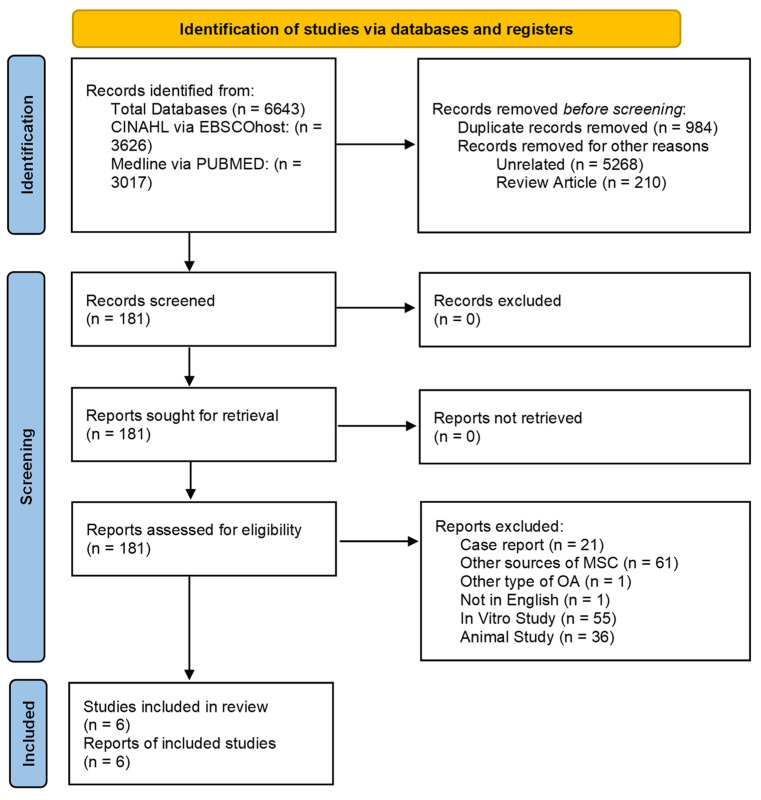
Flow chart showing article selection process.

The article screening process occurred in several phases. Initially, any article with a title that did not meet the inclusion criteria was excluded. Then, the abstracts of the remaining articles were evaluated, and those failing to meet the inclusion criteria were excluded. In the final phase, the full texts of the remaining articles were thoroughly assessed and included in our study if they met the inclusion and exclusion criteria. All authors participated in the selection and data extraction process, and any disagreements were resolved through consensus. A flowchart summarizing the article selection process and reasons for exclusion is presented in Figure 1.

### 2.5. Data Extraction

The data from the relevant studies were individually extracted, detailing both the study design and outcomes. Key aspects of the study designs included the sample size, subject characteristics, methods of MSC collection and processing, and the duration of follow-up. The reported outcomes encompassed safety, clinical efficacy, and radiological assessments.

## 3. Results

### 3.1. Study Design and Patient Demographics

Six studies met the inclusion and exclusion criteria, which included a total of 97 patients with 134 knees who received hUC-MSCs. Four studies were nonrandomized [10,11,12,13], while the other two were randomized [14,15]. For the Kellgren–Lawrence (KL) classification, all six studies included patients who suffered from knee OA KL classifications I to IV. Follow-up periods ranged from 3 to 48 months.

### 3.2. hUC-MSC Preparation

All studies explained the method of processing their hUC-MSCs, with MSC criteria in accordance with the International Society for Cellular Therapy (ISCT) guidelines [16]. 

### 3.3. Administration Method

In terms of number of injections, 3 studies used a single-injection technique to deliver the hUC-MSCs into the knee [11,12,15], while the other studies divided the hUC-MSCs into 2 injections, with 1 study administering the injection within 1 month apart [10], while another study administered the injections 6 months apart [14]. Another study divided the MSCs into four injections in 1-week intervals [13].

All 6 studies used a different dose for their studies, with the doses of hUC-MSCs ranging from 10 × 10^6^ cells to 10 × 10^7^ cells. One study used body weight as a guide for the dose of hUC-MSCs [10]. Other studies used a fixed dose, as mentioned in Table 1. In addition, another study also injected hyaluronic acid (HA) during the injection of hUC-MSCs, which was repeated on the 2nd and 3rd weeks [11].

### 3.4. Adverse Effects

No serious adverse effects were observed in any of the studies. The most common adverse effect was knee effusion, which resolved spontaneously in all cases. Another common adverse effect seen was pain. One patient developed superficial vein phlebitis over the right short saphenous vein, which resolved spontaneously [10].

### 3.5. Functional Outcome

Among the 6 selected studies, there were multiple clinical parameters that were used to monitor clinical improvement, which included the visual analog scale (VAS) [11,12,13,14,15], the Western Ontario and McMaster Universities Osteoarthritis Index (WOMAC) [11,12,13,14], the Knee Injury and Osteoarthritis Outcome Score (KOOS) [10,15], the International Knee Documentation Committee (IKDC) [11], the SF-36 questionnaire [12,14], and the SF-12 questionnaire [13].

Out of 5 studies, 1 study reported VAS improvement with statistical significance [12]. In another study, a statistically significant result was also observed only in the repeated-injection group (MSC-2) compared to the single-injection group and control group [14], while VAS was statistically significant initially but was not sustained by 12th month [11]. While for the other study [15], there were some improvements; however, they were not statistically significant.

As for the WOMAC scores, 3 studies reported improvement with statistical significance [11,12,14]. Furthermore, there was a steady improvement in the WOMAC scores over the whole study period in MSC-2 (2 injections) as compared to MSC-1 (single-injection group) [14].

In two studies [10,15], there was a significant clinical improvement in the KOOS parameter. A significant improvement in the KOOS parameter was observed after 4 years of follow-up [10]. In another study, there was an improvement up to 8 weeks; however, these clinical improvements were not sustained [15].

In terms of the IKDC parameter, a statistically significant improvement was observed, with the mild OA group showing sustained improvement till study endpoint [11].

The author did not provide statistically relevant data in one of the studies [13]; however, there was generally a significant improvement in all parameters measured.

### 3.6. Radiological Outcome

There was no standardized timing or outcome measurements in any of the studies regarding radiological outcomes. Each clinical trial used different protocols and timing intervals to measure improvement, as mentioned in Table 2.

The severity of abnormal changes either disappeared or shifted to a milder form, which was statistically significant in one of the studies [10]. In another study, the measurement using MOAKS in 14 articular subregions showed a mixed result, with T2 values decreased in 9 of 14 regions; however, only 1 region was statistically significant, and an increase in cartilage thickness was observed in 10 of 14 regions, but only significant in 5 regions [12]. In other studies [11,14,15], the MRI outcomes were not statistically significant.

## 4. Discussion

The injection of hUC-MSCs is safe, as demonstrated by this and other systematic reviews, which included other types of MSCs, commonly bone marrow and adipose tissue [17,18,19,20]. The most common adverse effect of MSC knee injection was knee swelling or effusion, which spontaneously resolved without intervention. The adverse effects may be related to the dose of MSCs, which was demonstrated by another systematic review, where a higher dose of MSCs caused more adverse events [18]. Even though MSCs are generally regarded as having low immunogenicity, they can still trigger an immune reaction [21]. Another point of concern is about the neoplastic potential in the joint due to stem cells’ multidirectional growth potential. Up to this point in time, according to current data, no adverse effects in terms of joint deformity, tumor formation, or death have been reported [22]. Long-term follow-up studies showed that serum tumor markers did not increase from before to 3 years after MSCs therapy [23].

Based on the results of this systematic review, hUC-MSCs are effective for treating knee OA, from mild to severe forms of knee OA. HUC-MSC administration could improve functional outcomes, or VAS, WOMAC, KOOS, and IKDC scores, with a persistent improvement that may be archived even beyond 48 months, which may signify a functional disease-modifying therapy [10]. It is possible that the effects of hUC-MSCs are more significant in lower grades of knee OA [11], whereas the paracrine activity of MSCs would be more effective in milder knee OA [19].

Multiple injections of hUC-MSCs may be more effective compared to a single dose, as, in one study, the MSC-2 injection group showed more significant improvements compared to the single-injection group [14].

Radiological outcomes demonstrated mixed results. There was an improvement in cartilage thickness; however, this was generally not significant, which was also observed by another network meta-analysis of different sources of MSCs [22]. Clinical outcomes may not correlate with MRI findings, with hUC-MSCs still being useful for symptomatic treatment rather than restoration to normal knee articular cartilage and anatomy. The ‘unrelated’ outcome of clinical and radiological findings may be due to a misconception in the understanding of MSCs. MSCs were named after a group of cells that could be isolated and cultured while maintaining the potential to be induced into other types of mesoderm cells. Thus, unfortunately, the term is being used to infer that these cells differentiate into regenerating tissue-producing cells. The term “Medicinal Signaling Cells” has been recommended to better describe these cells’ ability to target sites of injury or disease and release bioactive factors that are both immunomodulatory and regenerative, functioning as therapeutic agents [24]. Upon activation by injury or other methods, MSCs secrete several bioactive molecules that create a regenerative microenvironment by establishing a powerful trophic field [21].

Comparing multiple sources of MSCs, hUC-MSCs were the most effective in improving function in one network meta-analysis [22].

## 5. Limitations

There are a few limitations to this systematic review. The sample sizes were small in all the clinical trials. The dose of hUC-MSCs varied among studies, with some studies adding other adjuncts to the knee injection. The clinical trials in these studies lacked uniformity in monitoring the clinical and radiological outcomes including the MRI parameter, resulting in interpretation and data analysis difficulties. The study periods were also relatively short and with variable intervals of monitoring. Most of the study periods in this systematic review were less than 1 year. A longer duration is needed to see any possible long-term adverse effects and clinical outcomes. All these differences may have led to differences in evaluating the therapeutic effects of hUC-MSCs in the treatment of knee OA. More clinical trials with a longer follow-up period and larger patient numbers are needed to prove the safety and efficacy of hUC-MSCs in treating knee OA.

## 6. Conclusions

The intra-articular injection of hUC-MSCs is safe and improves pain and functional outcomes in the treatment of knee OA. Multiple injections of hUC-MSCs show better results compared to the single-injection technique. The use of hUC-MSCs for the treatment of knee OA may be the mainstay of therapy in the future.

## Figures and Tables

**Table 1:** Study design, subjects, procedure, and dose.

Reference	Study Design	Subjects	Procedure/Dose
Samara et al. [10]. Date published: 2022 University of Jordan, Amman, Jordan	Phase I/II Non-randomized	Number of patients: 16 (6♂ and 10♀) Number of knees: 25 knees (13 left knees and 12 right knees) Age: 42–73 years KL grade: III: 13 IV: 3 Follow-up period: 48 months	Number of injections: 2 Interval of injections: 1 month apart Dose: 1.2 × 10^6^ of hUC-MSCs per kg body weight Mean dose: 86.19 × 10^6^ (in 2 injections)
**Reference**	**Study Design**	**Subjects**	**Procedure/Dose**
Matas et al. [14]. Date published: 2018 University of Los Andes Clinical Centre, Santiago, Chile	Phase I/II Randomized (triple blind) Patients divided into 3 groups (1:1:1 ratio)	Number of patients: 26 (10♂ and 16♀) Control: 8 MSC 1: 9 MSC 2: 9 Age: 40–65 years (mean age 56 years) KL grade: II: 18 III: 11 Follow-up period: 52 weeks	Number of injections: 2 Control: 2 HA injections MSC-1: 1 hUC-MSCs, 1 placebo MSC-2: 2 hUC-MSCs Interval injections: 6 months apart Dose: 20 × 10^6^ of hUC-MSCs Placebo: 5% AB plasma in 3cc saline
	(1) Control group: HA at baseline and 6 months (*n* = 8); (2) MSC-1 group: hUC-MSCs only at baseline, placebo at 6 months (*n* = 9); (3) MSC-2 group: hUC-MSCs at baseline and 6 months (*n* = 9).		
**Reference**	**Study Design**	**Subjects**	**Procedure/Dose**
Soltani et al. [15]. Date published: 2018 Iran University of Medical Sciences, Tehran, Iran	Randomized (double-blind) 20 randomized into 2 groups (*n* = 10 in each group)	Number of patients: 20 Control: 10 MSC: 10 Age: - KL grade II–III: 18 IV: 2 Follow-up period: 24 weeks	Number of injections: 1 Dose: 50–60 × 10^6^ of hUC-MSCs Placebo: 10cc normal saline
**Reference**	**Study Design**	**Subjects**	**Procedure/Dose**
Dilogo et al. [11]. Date published: 2020 Cipto Mangunkusumo Hospital, Jakarta, Indonesia	Non-randomized	Number of patients: 29 (17♂ and 12♀) Number of knees: 57 knees Age: 58.3 ± 9.68 years KL grade I–II: 33 III–IV: 24 Follow-up period: 12 months	Number of injections: 3 Interval: 1 week apart Dose: 10 × 10^6^ of hUC-MSCs and 2 mL HA 2nd and 3rd week: 2 mL HA
**Reference**	**Study Design**	**Subjects**	**Procedure/Dose**
Gunay et al. [12]. Date published: 2022 Kayseri City Education and Research Hospital, Turkey	Non-randomized	Number of patients: 10 (3♂ and 7♀) Age: 58.2 ± 10.0 years KL grade: - Follow-up period: 12 months	Number of injections: 1 Dose: 1 × 10^8^ of hUC-MSCs
**Reference**	**Study Design**	**Subjects**	**Procedure/Dose**
Ao et al. [13]. Date published: June 2023 Centre for Joint Surgery, Southwest Hospital, Third Military Medical University (Army Medical University), Chongqing, China	Phase 1 Non-randomized	Number of patients: 14 (4♂ and 10 ♀) Age: 58.29 years ± 8.99 KL grade: Grade 2: 10 Grade 3: 4 Follow-up period: 3 months	Number of injections: 4 Interval: 1 week apart Dose: 6 × 10^7^ of hUC-MSCs (divided into 4 injections)

**Table 2:** Safety, clinical, and radiological outcomes.

Reference	Safety	Clinical		Radiological	
Samara et al. [10]. Date published: 2022 University of Jordan, Amman, Jordan	No serious adverse events Mild Mild pain (grade 1–3): 7 Moderate pain (grade 4–5): 5 Knee effusion: 1 Superficial vein (right short saphenous vein) phlebitis: 1 All treated conservatively	Parameter (baseline, 6, 12, 48 months)		Timing: baseline, 6 and 12 months Parameter: 6 (cartilage loss, osteophytes, BM lesions, effusion, synovitis, subchondral sclerosis) into 4 groups (none, mild, moderate, severe) The severity of abnormal changes either disappeared or shifted to a milder form, which was statistically significant (*p* = 0.01); For subchondral sclerosis subtype, improvement was more significant (*p* = 0.0001)	
		(1) KOOS - Significant improvement in KOOS at 6, 12 and 48 months (*p* = 0.0001)			
**Reference**	**Safety**	**Clinical**		**Radiological**	
Matas et al. [14]. Date published: 2018 University of Los Andes Clinical Centre, Santiago, Chile	No serious adverse effects	Parameter (baseline, 6, 12 months)		Timing: baseline, 24 and 48 weeks Parameter: Whole-Organ Magnetic Resonance Imaging Score (WORMS) No significant change from baseline was noted (*p* = 0.15)	
	(1) Acute knee effusion:	(1) WOMAC (Spanish validated version) (2) VAS (3) SF-36 questionnaire (4) OMERACT-OARSI Responder Index Criteria			
	MSC-1 and MSC-2 (33%) compared to HA group (22%) (*p* = 0.99) MSC-2 2nd injection (44%) compared to HA group (37.5%) (*p* = 0.99)	In MSC-2 group (compared to HA and MSC-1) at 12 months			
		(1) WOMAC - Total: *p* = 0.04 - Pain: *p* = 0.04 - Stiffness: *p* = 0.14 - Function: *p* = 0.08 (2) VAS: *p* = 0.02 (3) No changes in SF-36			
	(2) Pain: no statistical difference (0.99)	MSC-1 group: Improvement until month 9, then similar to control (HA group after second dose) MSC-2 group: Improvement until study endpoint			
**Reference**	**Safety**	**Clinical**		**Radiological**	
Soltani et al. [15]. Date published: 2018 Iran University of Medical Sciences, Tehran, Iran	No serious adverse effects Four patients in the MSC group had increased local pain and mild effusion	Parameter (baseline, 2, 8, 24 weeks)		MRA at 0 and 24 weeks	
		(1) VAS: *p* = 0.401 (2) ROM: statistically significant between pre-intervention and 24 weeks (*p* = 0.002) compared to not statistically significant in control group (3) KOOS: until 8 weeks *p* = 0.05 However, these clinical improvements were not sustained until 24th week, *p* = 0.483		(1) Cartilage thickness (28 point) (2) Presence of synovial hypertrophy (3) Spur (4) Erosion (5) Medial/lateral meniscus (6) ACL injuries	
				In MSC group, significant increase in cartilage thickness in some area.	
**Reference**	**Safety**	**Clinical**		**Radiological**	
Dilogo et al. [11]. Date published: 2020 Cipto Mangunkusumo Hospital, Jakarta, Indonesia.	No serious adverse effects	Parameter: (baseline, 6, 12 months)		MRI frequency: 3 (baseline, 6 and 12 months) Parameter: quantitative T2 mapping of medial and lateral cartilage of knee The result of T2 mapping showed a varied result with no statistically significant differences	
		(1) VAS: Both groups showed reduction, however only significant in severe OA group at 6th month (*p* = 0.035), however not sustained until 12th month (*p* = 0.153). Mild OA group: 12th month (*p* = 0.158) (2) IKDC: Significant improvement in both groups at 6th month (mild OA: *p* = 0.001, severe OA: *p* = 0.008), however only mild OA group maintained until 12th month (mild OA: *p* = 0.038, severe OA: *p* = 0.28) (3) WOMAC: Mean reduction was statistically significant in both groups - Mild OA: 6th month: *p* = 0.003 12th month: *p* = 0.071 - Severe OA: 6th month: *p* = 0.003 12th month: *p* = 0.044			
**Reference**	**Safety**	**Clinical**		**Radiological**	
Gunay et al. [12]. Date published: 2022 Kayseri City Education and Research Hospital, Turkey	No allergic or adverse reaction was noted 3 patients showed mild effusion	Parameter: (baseline, Day 21, Day 42, 3rd month, 6th month, 12th month) Improvement in Month 12 assessment (compared to pre-injection)		MRI frequency: 2 (baseline, 12 months) Parameters: 1) T2 cartilage mapping and cartilage thickness (MRI osteoarthritis knee score (MOAKS) in 14 articular subregions) T2 values after injection decreased in 9 of 14 regions measured, with only 1 region significant For cartilage thickness, an increased value was observed in 10 of 14 regions but only significant in 5 regions	
		(1) VAS: *p* = 0.000 (2) WOMAC: *p* = 0.007 (3) Lequesne: *p* = 0.239 (4) SF-36: *p* = 0.032			
**Reference**	**Safety**	**Clinical**		**Radiological**	
Ao et al. [13]. Date published: June 2023 Center for Joint Surgery, Southwest Hospital, Third Military Medical University, Chongqing, China	No serious adverse effects All adverse effects are transient	(1) Clinical			
		Parameter: (baseline, 12 weeks) (all data are presented as median (interquartile range))			
			Pre-operative		Post-operative
	(1) Post-injection pain: 4 patients (2) Swelling: 1 patient (3) Fever: 3 patients (4) Numbness: 1 patient (5) Stiffness: 1 patient (6) Dizziness: 1 patient	VAS score	6.0 (4.5, 8.3)		3.5 (2.0, 5.0)
		WOMAC score	26.0 (21.0, 37.0)		8.5 (7.0, 12.75)
		SF-12	39.0 (35.8, 42.3)		46.0 (44.0, 48.3)
		(2) Radiological			
		Parameter: (baseline, 12 weeks) (all data were presented as median (interquartile range)			
			Pre-operative		Post-operative
		MOCART score	42.0 (34.0, 48.0)		58.0 (49.0, 68.3)

## Data Availability

The data are provided within the article and are available from the corresponding author upon request.
